# Software-Assisted Depth Analysis of Optic Nerve Stereoscopic Images in Telemedicine

**DOI:** 10.1155/2016/7603507

**Published:** 2016-04-14

**Authors:** Tian Xia, Shriji N. Patel, Ben C. Szirth, Anton M. Kolomeyer, Albert S. Khouri

**Affiliations:** The Institute of Ophthalmology and Visual Science, Rutgers New Jersey Medical School, Newark, NJ 07103, USA

## Abstract

*Background*. Software guided optic nerve assessment can assist in process automation and reduce interobserver disagreement. We tested depth analysis software (DAS) in assessing optic nerve cup-to-disc ratio (VCD) from stereoscopic optic nerve images (SONI) of normal eyes.* Methods*. In a prospective study, simultaneous SONI from normal subjects were collected during telemedicine screenings using a Kowa 3Wx nonmydriatic simultaneous stereoscopic retinal camera (Tokyo, Japan). VCD was determined from SONI pairs and proprietary pixel DAS (Kowa Inc., Tokyo, Japan) after disc and cup contour line placement. A nonstereoscopic VCD was determined using the right channel of a stereo pair. Mean, standard deviation, *t*-test, and the intraclass correlation coefficient (ICCC) were calculated.* Results*. 32 patients had mean age of 40 ± 14 years. Mean VCD on SONI was 0.36 ± 0.09, with DAS 0.38 ± 0.08, and with nonstereoscopic 0.29 ± 0.12. The difference between stereoscopic and DAS assisted was not significant (*p* = 0.45). ICCC showed agreement between stereoscopic and software VCD assessment. Mean VCD difference was significant between nonstereoscopic and stereoscopic (*p* < 0.05) and nonstereoscopic and DAS (*p* < 0.005) recordings.* Conclusions*. DAS successfully assessed SONI and showed a high degree of correlation to physician-determined stereoscopic VCD.

## 1. Introduction

Glaucoma is one of the most prevalent causes of blindness worldwide. Once characteristics damage to the optic nerve occurs, the loss of vision and visual field is irreversible [1]. An essential step in identification of glaucomatous optic neuropathy is assessment of the optic nerve head (ONH) for structural changes. Increases in cup-to-disc ratio (CDR) may represent development or progression of glaucoma thereby warranting further investigation [2]. Reliable assessment of the ONH during telemedicine screening is difficult for myriad reasons. For example, the three-dimensional aspect of CDR is poorly appreciated from two-dimensional images obtained with nonmydriatic cameras. Three-dimensional evaluation of the ONH has been shown significantly more accurate in comparison to traditional two-dimensional pictures [3]. In addition, discrepancies in observer training and experience evaluating the ONH can lead to significant variability in patient referral patterns [4]. Software guided optic nerve assessment can assist in automating the process and reduce interobserver disagreement, regardless of the level of ophthalmic training [5].

With the ever increasing number of people diagnosed with glaucoma and insufficient number of glaucoma specialists worldwide [6], the use of software-assisted CDR assessment by ancillary medical professionals with varying levels of experience during remote telemedicine screenings may help to triage patients appropriately and thereby decrease the overall burden of disease. In the following, we characterize the ability of depth analysis software to assess CDR from simultaneous stereoscopic images of normal optic nerves. We then conduct comparisons with physician-determined stereoscopic and nonstereoscopic CDR to establish data correlation. This represents the first step in validating the use of this software in screening and telemedicine applications.

## 2. Materials and Methods

Consecutive normal subjects were prospectively enrolled in this pilot study. A normal exam was defined as healthy anterior and posterior segments, refractive errors between −6 and +4 diopters, noncontact intraocular pressure (IOP) <21 mm Hg, normal optic nerve head appearance with a CDR <0.6 (upper limit of population 95% confidence interval), and no signs of glaucomatous neuropathy. The Rutgers University New Jersey Medical School Telemedicine Outreach Program Services collected demographic/clinical patient data, performed ocular examination, and obtained simultaneous stereoscopic optic nerve images during community screenings in Essex County, NJ, from 2011 to 2012. We certify that all applicable institutional and governmental regulations concerning ethical use of human volunteers were followed during this research.

Nondilated images were acquired by a single user (BCS) using a Kowa 3Wx nonmydriatic simultaneous stereoscopic retinal camera (Tokyo, Japan) with a resolution of 12.3 megapixels and a field of view of 22.5 degrees. No image enhancement or compression was performed. Images were assessed following an established protocol [7]. All images were viewed on a single high-resolution monitor (1080 pixels) in a darkened room. Stereoscopic image quality was determined based on the ability to acquire satisfactory right and left image pairs and to view the nerve stereoscopically.

Vertical cup-to-disc ratio (VCD) was determined from a stereoscopic pair. Stereoscopic viewing was performed using a Berezin stereo viewer of a stereoscopic image pair at a distance of 18 inches. On a different day, in a randomized order and unaware of the software grading, VCD was determined from a single image (nonstereoscopic using the right channel image), then again using the depth analysis software.

**Figure 1:**
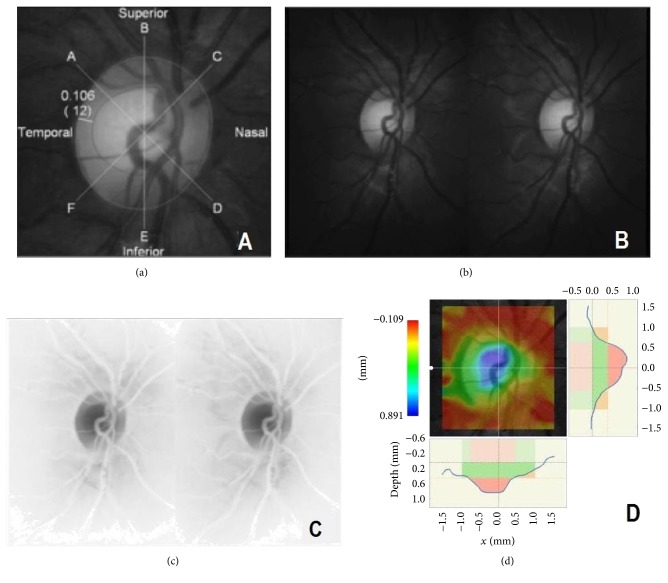
Starting with screening images taken by the nonmydriatic Kowa retinal camera, the CDR was assessed nonstereoscopically to place rim and disc contours (a) with assistance of the green channel (b) and negative image (c). Software generated depth analysis map (d).

A novel proprietary pixel depth analysis software (Tokyo, Japan) was employed for the software analysis. The software registers the stereo image pair and makes quantitative depth calculations based on right and left image channel disparity measurement. This software was used to determine VCD after disc and cup contour line manual placement by one physician operator (SNP; Figure 1(a)). A total of 16 control points were used by the software to generate a disc (8 points) and a cup margin (8 points) contour line. In order to improve the quality of line positioning, we relied on stereoscopic viewing with the Berezin viewer during control point placement. In addition, we used the green channel (550 nm; Figure 1(b)) to best appreciate the neural rim during cup determination. The contrast provided by the optic nerve “negative” image was used to determine disc margins and aided in disc contour line placement (Figure 1(c)). The software generated a depth analysis map for each stereo pair and calculated VCD (Figure 1(d)).

Composite nonstereoscopic, stereoscopic, and software generated VCD values were expressed as means ± standard deviation (SD). Student's *t*-test was used to compare the mean VCD values. The measurement of agreement between observations was quantified using the intraclass correlation coefficient (ICC). Statistical analysis completed using IBM SPSS (Version 17, NY, USA), with *p* < 0.05 signifying statistical significance.

## 3. Results

A total of 32 stereoscopic ONH images from 32 subjects were included in the analysis. Mean ± SD age was 40 ± 14 years (range, 21–60 years). There were 28 (88%) females and four (12%) males. Fourteen (44%) participants were Caucasian, 13 (41%) Hispanic, three (9.4%) Asian, and two (6.3%) African American. Iris color was brown in 23 (72%) participants, hazel in five (16%), and blue in four (12%) subjects. Image stereoscopic quality was adequate in all subjects for high fidelity analysis by clinician and software.

The mean VCD with stereoscopic images was 0.36 ± 0.09 (95% CI 0.33–0.40), nonstereoscopic images was 0.29 ± 0.12  (95% CI 0.25–0.32), and depth analysis software was 0.38 ± 0.08 (95% CI 0.35–0.41). The difference between stereoscopic and depth analysis software-assisted recordings was not significant (*p* = 0.45). The difference in mean VCD was significant between nonstereoscopic and stereoscopic (*p* < 0.05) and nonstereoscopic and depth analysis software (*p* < 0.005) recordings. The ICC between stereoscopic and software-assisted recordings was 0.88 (strong), between nonstereoscopic and stereoscopic recordings was 0.70 (moderate-strong), and between nonstereoscopic and software-assisted recordings was 0.56 (moderate).

## 4. Discussion

In this pilot study, we set out to assess the ability of a novel depth analysis software to reliably measure CDR by comparing it to physician-determined CDR assessed from high quality simultaneous stereoscopic images. We demonstrated that this depth analysis software accurately assessed stereoscopic optic nerve images and showed no statistically significant difference from physician stereoscopic assessment of VCD. There was also a high degree of agreement between software and physician stereoscopic assessment in normal optic nerves as demonstrated by the intraclass correlation coefficient.

The importance of teleocular health screening in at-risk, low access populations is widely accepted and software-assisted screening permits thorough, efficient evaluation of ocular health [7, 8]. However, at present there is no standardized method for evaluating the ONH during telescreenings. Furthermore, glaucomatous eyes require long term and frequent monitoring of ONH changes. Software-assisted stereoscopic imaging not only may be helpful in evaluation of ocular health but also may allow for efficient long term monitoring. There is a large amount of literature demonstrating limited interobserver and intraobserver reproducibility in regard to optic disc assessment [9–11]. Nonetheless, stereoscopic ONH imaging has been shown superior to monoscopic imaging and, with low-cost Internet transmission, was better perceived and interpreted than monoscopic images [12]. Vessani et al. demonstrated that stereoscopic images of several cameras have better diagnostic performance than subjective assessment of ONH by general ophthalmologists [13]. Other studies have also demonstrated the improved detection of at-risk eyes between stereoscopic imaging and nonstereoscopic imaging with higher CDR in all regions of the ONH and even increased interobserver agreement using stereoscopic imaging compared with monoscopic assessments [3, 14, 15]. These studies suggest that stereoscopic imaging and depth software analysis could supplement the clinical exam in glaucoma detection during remote screenings and telemedicine efforts.

Previous studies have shown software ability to analyze stereo image pairs of a retinal fundus and generate ONH parameters with some degree of consistency. These have shown reliability between depth analysis software assessment of CDR and Heidelberg Retina Tomograph (HRT) [16, 17]. In addition, HRT has been shown to have superior sensitivity to the average ophthalmologist in detecting glaucomatous optic nerve changes [18]. More interestingly, Wollstein et al. showed that depth analysis using HRT image analysis is more sensitive than clinical assessment of stereoscopic images in differentiating healthy and early glaucomatous ONH [19]. Similar studies tested other methods of automated CDR assessment including edge detection, active contour modeling, and pixel segmentation in comparison to expert ophthalmologist assessment with similar results [20–22]. Automated determination using optical coherence tomography has also shown promise in comparison to manual assessment [23]. Levels of agreement in CDR estimations in above-mentioned studies are comparable to those reported in our study.

While our study shows promise towards clinical, screening, and telemedicine applications, there are a few inherent limitations, primary of which is the need for contour line placement in order for the software to perform ONH measurements. This step may require some experience and may contribute to variability in characterizing ONH parameters. However, this requirement is similar to the HRT, and a recent study showed good agreement in stereometric parameters between our software and the HRT platform [24]. In order to minimize this potential source of error, we relied on stereoscopic viewing during contour line placement. In addition, we used the green channel to best appreciate the neural rim during cup determination and a negative image to best depict the disc margin during disc contour line placement. Another limitation is the relatively young age of the population compared to patients with glaucoma; however, our data validated the use of this software for CDR assessment in normal eyes over a wide range of ages. Additional studies validating the use of this promising technology in the elderly and myopes may be helpful prior to applying it to patients with glaucomatous optic neuropathy.

In summary, the new depth analysis software was able to reliably calculate VCD from a digital stereoscopic image pair in normal patients and strongly correlated with the clinician determined stereoscopic ONH analysis. The preliminary results of this study were presented at the ARVO conference in Fort Lauderdale, Florida, in May 2012 [25]. The ability to accurately perform depth analysis from a single stereoscopic image pair could have several clinical applications especially in remote screening efforts and telemedicine applications. Despite the limitations and additional studies required to determine the full clinical application of this software, we believe that the most immediate use of this software would be to train nonphysician staff to place the optic nerve contour lines and allow the software to reliably estimate CDR in locations where physicians may not be available to provide real time feedback. This analysis would be used to triage patients appropriately. An additional clinical utility of this software may stem from its ability to characterize the neural rim, which may be helpful in following glaucoma patients over time.
